# Synthesis and characterization of butyl acrylate-co-poly (ethylene glycol) dimethacrylate obtained by microemulsion polymerization

**DOI:** 10.1080/15685551.2020.1739506

**Published:** 2020-03-16

**Authors:** Abraham G. Alvarado, Martin Rabelero, Jacobo Aguilar, Jorge Flores Mejia, Francisco J. Moscoso Sánchez

**Affiliations:** a Departamento de Ingeniería Mecánica Eléctrica, Centro Universitario de Ciencias Exactas e Ingenierías, Universidad de Guadalajara, Guadalajara, México; b Departamento de Ingeniería Química, Centro Universitario de Ciencias Exactas e Ingenierías, Universidad de Guadalajara, Guadalajara, México; c Departamento de Ciencias Tecnológicas, Centro Universitario de la Ciénega, Universidad de Guadalajara, Ocotlán, México; d Departamento de Química, Centro Universitario de Ciencias Exactas e Ingenierías, Universidad de Guadalajara, Guadalajara, México

**Keywords:** n-butyl acrylate, poly(Ethylene glycol) dimethacrylate, copolymers, microemulsion, gel

## Abstract

The synthesis and characterization of copolymers of n-Butyl Acrylate (BA) and Poly(ethylene glycol) dimethacrylate (PEGDMA) were realized by microemulsion. In this synthesis, the relation of PEGDMA 10, 20, 30, 40 and 50% wt with respect to BA was changed. The copolymers obtained were characterized by the determination of conversions (gravimetry), infrared spectroscopy: Fourier transform (FTIR), dynamic light scattering (DLS), thermogravimetry (TGA) and differential scanning calorimetry (DSC). The results confirmed the synthesis of BA-co – PEGDMA copolymers by the identification of characteristic FTIR bands and which determined the glass transition temperature of the copolymers. The conversions were found in the range of 85% to 90%. Within the stability of the produced latex, it was observed that at 10% and 30% wt. of PEGDMA the systems were stable, but when more PEGDMA was added up 40% to 50% wt., the system became unstable. The stability of produced latexes depends on the PEGDMA contents and this must be less than 30% wt.; meanwhile the PEGDMA content greater than 30% wt. leads to unstable latexes, forming clots. Copolymers showed single glass transition temperatures between −53.37°C and −16.58°C, depending on the composition of PEGDMA in the copolymers. Resulting in the different arrangements of units of PEGDMA along in the chain affected the thermal properties of the final copolymers.

## Introduction

Nowadays, microemulsion polymerization is an important technique to prepare highly stable latex-containing small particles of less than 100 nm and polymers with molecular weights exceeding one million [1,2]. This growing interest is due to the fact that there are many potential benefits in a variety of technologies [3,4]. A number of these investigations have used vinyl [5,6] monomers and glycols [7,8]. The versatility that exists is such that it can make a variability in structures and form different synthesis methodologies. The polymeric nanoparticles with complex structures varied, with attractive functions; such as being a different approach for nanoparticle fabrication. Example of this, it is a self-assembly-combined copolymer with nanoskiving. This fabrication method allows an easy experimental procedure that does not require specialty fabrication equipment. This is advantageous because it is expected to result in nanoparticles with homogeneous and optimized surfaces [9,10]. Applications in many fields of investigation with these monomers had been possible in agriculture, medical, pharmaceutical, and textile industries. In medicine, the use of micellar solutions presents application advantages and disadvantages. Micellar solutions with surfactants have been one of the popular methods for the solubilization of hydrophobic drugs; however, surfactants with bigger molecular masses present high critical micelle concentration with low stabilities. Instead, surfactants of low molecular masses show general advantages such as higher stability, tailorability, greater cargo capacity, non-toxicity and controlled drug release [11]. Kampmann et al. [12] reported the first synthesis and characterization of polymeric nanoparticles in a microemulsion polymerization based on bifunctional poly(2-oxazoline) macromonomers. The concept involves the use of a bifunctional amphiphilic polymer surfactant that carries multiple acrylate groups in the hydrophobic block to serve as a macromonomer during the formation of the nanoparticles. Particle sizes can vary between 20 and 75 nm depending on the amount of core cross-linker used. Furthermore, to demonstrate the versatility of this approach an amino endgroup of the poly(2-oxazoline) macromonomer was used for post-analogous modification of the particles with folic acid, a RGD-peptide derivative, and fluorescein. González-Ayón et al. [13] prepared nanohydrogels synthesized by surfactant-free emulsion polymerization with a core-shell structure. The core was made of poly(n-vinylcaprolactam) and the shell of poly (ethyleneglycol) methacrylate and either n-vinylpyrrolidone or 2-methacryloyloxybenzoic acid. The nanohydrogels produced were used to encapsulate 5-Fluorouracil (an anticancer drug) optimizing the drug release of different pH and temperature values by adjusting the transition temperature of the materials near to physiological temperature values. Mustafa et al. [14] reported the synthesis of methyl methacrylate/hydroxyethyl methacrylate (MMA/HEMA) with modifying the feeding and composition of monomers in the microemulsion. Also, three different drugs were loaded in the copolymeric nanospheres, resulting in homogeneous stable latex. The influence of variation of HEMA content in the composition, the average particle size, turbidity, surface tension, and zeta potential were investigated. As well as, studying the relation of copolymer/drug the effect of drug hydrophobicity, drug content on the loading into a copolymer and the in vitro drug release through a simulated intestinal fluid (pH 7.4) and in simulated gastric fluid (pH 1.2), at 37 ± 0.5°C. Ovando-Medina et al. [15] researched the effects of monomer addition rate and surfactant concentration on the kinetics, particle size, copolymer molecular weight, and composition in the semicontinuous heterophase copolymerization of vinyl acetate and butyl acrylate and compared against the results of the seeded semicontinuous process. Jia et al. [16] investigated the effect of polyethylene glycol (PEG) as a molecular crowding agent on reducing template consumption in the preparation of molecular imprinted polymers (MIPs). The polymerization was realized in situ, used acrylamide as a functional monomer and ethylene glycol dimethacrylate as the cross-linking monomer. A mixture of PEG-Tetrahydrofurane-toluene as a porogen was used. Demonstrated that the PEG has a crowding effect in the MIP monoliths preparation with good column permeability, imprinting effect and a significant reduction in the consumption of the template during the MIPs preparation. Tripathi et al. [17] applied an efficient Monte Carlo (MC) method to simulate the reaction kinetics and molecular structure progress during free-radical, bulk copolymerizations of a homologous series of methacrylate monomers with ethylene glycol dimethacrylate (EGMA). The researchers determined that the extent to which the n-alkyl ester side chains of methacrylate monomers negatively affect the ability of chain-end radicals; hence, propagating the pendant vinyl groups of EGDMA copolymerized into the polymer backbone. The investigators used a homologous series of methacrylate monomers to systematically change the lengths of the ester side chains while not having to deal with chain transfer to polymer backbone reactions which are commonly experienced when using acrylate monomers. Flores-Rojas and Bucio [18] carried out an investigation with silicone rubber (SR) modifying a binary graft of ethylene glycol dimethacrylate (EGDMA) and glycidyl methacrylate (GMA) using two different methods: (1) direct radiation and (2) using of AIBN as an initiator. The gamma-radiation method generated higher grafting yields, obtaining a maximum of up to 60.5% grafted copolymer, which is much higher than the 15.4% grafting yield obtained using AIBN. Another important aspect is that using the chemical method resulted in films with better flexibility than films obtained by radiation-induced graft polymerization. This change in flexibility is probably due to the differences in the film’s grafting degree, crosslinking, or degradation. Zhao et al. [19] demonstrated that copolymers composed of poly(ethylene glycol)- 4,5 lactic acid dimethacrylate (PEG-LA-DM) degradable and Poly(ethylene glycol) diacrylate (PEGDM) nondegradable, macromers are favorable scaffolds for engineering articular cartilage. There were no obvious differences observed amongst the studied copolymers at contrasting ratios (60/40 and 70/30) within the cell cartilage repair. Yet, further studies are required to research the potential of such tissue engineering. Oh [20] in 2011 conducted a review of recent advances in the synthesis and self-assembly of PLA-containing amphiphilic block copolymers (ABPs). The suggested approach would be to design and synthesize well-controlled PLA-based amphiphilic block copolymers (ABPs); as well as their bio-related applications, including drug delivery, imaging platforms of self-assembled nanoparticles, and tissue engineering of crosslinked hydrogels. In our study, the copolymerization of Butyl Acrylate (BA) onto Poly (ethyleneglycol) dimethacrylate (PEGDMA) was carried out to modify the physical properties of BA (base polymer) by adding different amounts of PEGDMA. The effects of adding the PEGDMA were studied in the copolymers synthesized through conversion (gravimetry), Fourier transform infrared, dynamic light scattering, thermogravimetry and differential scanning calorimetry.

## Experimental

### Materials

The Butyl acrylate and Poly (ethyleneglycol) monomers dimethacrylate from Aldrich of 99% pure (St. Louis, MO, USA) were purified by passing it through a DHR-4 column from PolyScience (Ontario, NY, USA). All other materials were used without further purification. Potassium persulfate from Fermont, ACS grade (Monterrey, Mex.) was used as an initiator and the anionic surfactant sodium dodecyl sulfate (SDS) as a stabilizer from Aldrich (St. Louis, MO, USA). The water used was de-ionized from Selectro Pura (Guadalajara, Mex.) in order to purge its oxygen content. The nitrogen gas was provided by Infra de Occidente (Guadalajara, Mex.). A dialysis bag: 11,200 by Dalton from Sigma Aldrich (St. Louis, MO, USA) was used to purify the synthesized copolymer.

### Procedure

**Figure 1.**
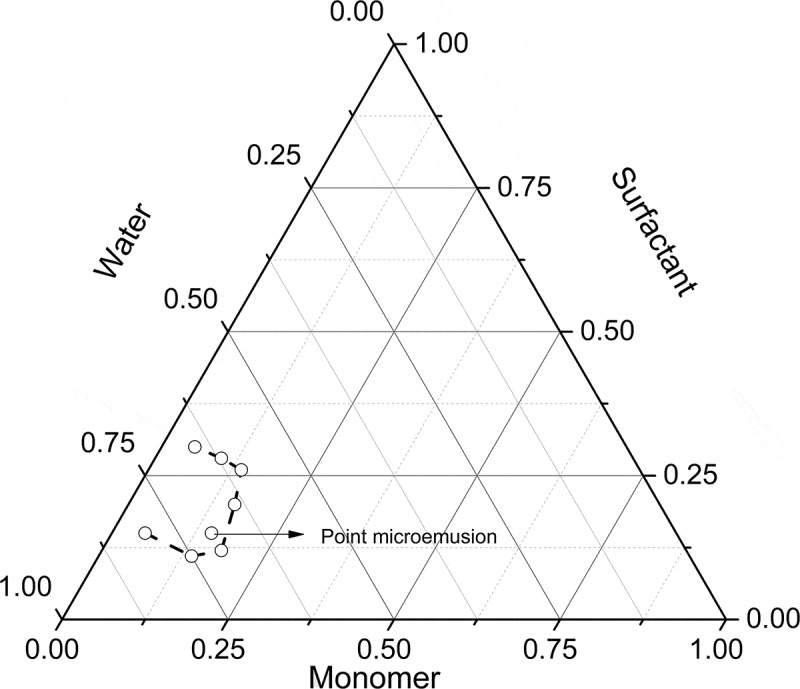
Phase diagram of system. Borders of the microemulsion region are given with bold lines and points

First, the concentrations at which this system forms microemulsions were determined. To achieve it, several weight ratios of surfactant-water-monomers (for a 50/50 wt. BA/PEGDMA) were prepared and heated to 60°C to identify the microemulsion regions. Figure 1 shows the pseudo-ternary phase diagram with the microemulsion region bounded by the dotted line. Afterwards, the concentration of the microemulsion was selected to carry out the polymerization reactions.

The polymerizations were done in a 250 mL glass reactor equipped with a reflux condenser and inlets for nitrogen gas, monomer feed, and sampling. Stirring was provided with a magnetic bar. Initially, 15 g of SDS and 70 g of water were loaded in the reactor, bubbled with nitrogen for 20 min, and heated to a reaction temperature of 60°C. Followed by the addition of 15 g of a monomers mixture (BA/PEGDMA) at different weight concentrations of BA (90%, 80%, 70%, 60% and 50% wt.). Finally, 1% wt initiator in relation to monomers was added. The reactions were done in a 120-min period.

### Polymer separation and characterization

The samples were withdrawn at given times in order to determine the conversion gravimetrically, and measure particle size, followed by a Fourier transformation infrared spectroscopy, which allowed the glass transition temperature, and thermo-gravimetric analysis. The polymer was purified in a dialysis bag from 11,200 Dalton. This membrane was effectively able to retain the polymer and the remaining components passed through it. The samples were suspended inside the membrane and submerged in hot water at 40°C for 15 days for complete polymer purification. The Z-Average particle size (Dpz) as a function of reaction time was measured at 25°C by dynamic light scattering in a Malvern Zetasizer ZS90 apparatus (Malvern, UK). The particle size standard operating subroutine procedure was used to estimate the particle-size distribution index (PSDI). In order to eliminate multiple scattering and to measure the monomer-free average particle size, the lattices were diluted with water up to 100 times. For the determination of the morphology and size of the obtained nanoparticles, 0.02 g of the latex dispersed in 30 mL distilled water, followed by 0.1 g of a 2% wt. phosphotungstic acid solution added as a staining agent to enhance the contrast between nanoparticles. A drop of the formed solution was deposited in a copper grid and allowed to dry for 24 h before the transmission electron microscope (TEM) observations. A JEOL 1010 transmission electron microscope (Peabody, MA, USA) was used at different magnifications. The copolymer formation an FTIR spectroscopy was used, and the spectra were obtained with a scientific thermo OMNIC 9 (Thermo Fisher Scientific, Madison, WI, USA) attenuating a total reflection at a 4 cm^−1^ resolution, with 32 scans verified at room temperature. For the glass transition temperature determination, a Q100 scanning calorimeter from TA Instruments (New Castle, USA), calibrated with indium was used. The Measurements were taken with a sample weight of 8–10 mg at a heating rate of 10°C min^−1^, from −80°C to 300°C using a nitrogen purge gas flow of 50 mL min^−1^. After the first heating scan, a second scan was conducted at a heating rate and similar conditions (10°C min^−1^). The glass transition temperatures were determined from the second scan by the reversible heat flow signal. The thermogravimetric analysis was carried out in a TA instrument Orchestration 7.2. TA Instruments (New Castle, USA), approximately 10 mg of samples were scanned ranging from 30°C to 600°C, and at a 10°C min^−1^ heating rate under the nitrogen purge gas flow rate of 20 mL min^−1^ to avoid sample oxidation.

## Results and discussion

The pseudo-ternary phase diagram with a weight ratio of 50/50 of BA to PEGDMA is described in Figure 1. The translucent microemulsion region is presented in phase diagram bounded by the dotted line. Out of this of the region represents the turbid and conventional emulsions based on visual observation. There was not a liquid crystalline structure observed with the use of a cross polarizer lens. According to Donescu et al. [21], the knowledge of the limit of solubilization of a monomer in the micellar aggregates is very important in interpreting the polymerization kinetics.

The presence of a vinyl group on the chain butyl acrylate can cause a rise to the chemical reactions with a vinyl group of poly (ethylene glycol) dimethacrylate, leading to a stable bonding of copolymer chains. The scheme of possible reaction between BA and PEGDMA is shown in Scheme 1. The reaction can vary over a wide range to obtain a product with a particular combination of some specific desirable properties.

**Scheme 1**. Reaction of BA/PEGDMA.

Table 1 shows a summary of the final conversions and times from the weight ratios of BA/PEGDMA, including the homo-polymers is reported. As one may appreciate, the conversions are greater than 85% within a 120-min period timing, for copolymer ratios 90/10, 80/20 and 70/30% by weight of BA/PEGDMA and homo-polymer of BA. These values are common for conversion and reaction time of microemulsions. However, for the relation of 60/40, 50/50% wt BA/PEGDMA and homo-polymers of PEGDMA, the conversions were 86%, 85% and 96%. Yet, the end time of reactions was of 10, 9 and 6 min. The pronounced increase in the time of polymerization can be discussed in terms of both increased particle concentration and the gel effect. The abrupt decrease in the polymerization time can be discussed in terms of the decrease in monomer concentration of BA at the reaction due to the increased fraction of polymer gel with increasing PEGDMA concentration. The major contributor to the gel content in this system is likely to be the PEGDMA monomer. The high PEGDMA content can lead the gel effect because the PEGDMA is more soluble in water, the same as the BA and the reaction was conducted in the aqueous phase. The effect has been caused by a decrease in the rate of termination of PEGDMA due to increased viscosity of the medium [22].

**Table 1.** Shows a summary of the final conversions and times

Relation BA/PEGDMA	Final time (min)	Final conversion (%)
100	120	98 ± 2
90/10	120	85 ± 2
80/20	120	81 ± 3
70/30	120	92 ± 4
60/40	10	86 ± 4
50/50	9	85 ± 2
0	6	96 ± 3

**Figure 2.**
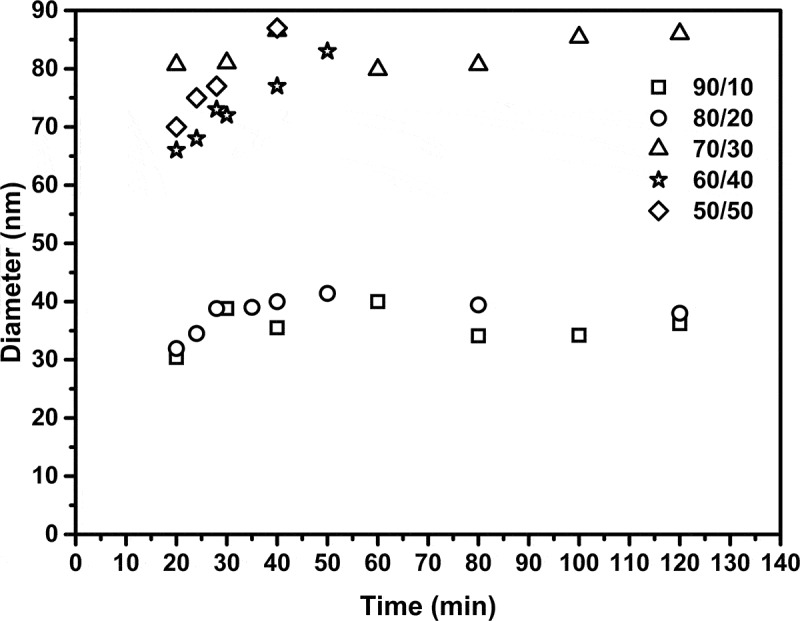
Particle size versus time

**Figure 3.**
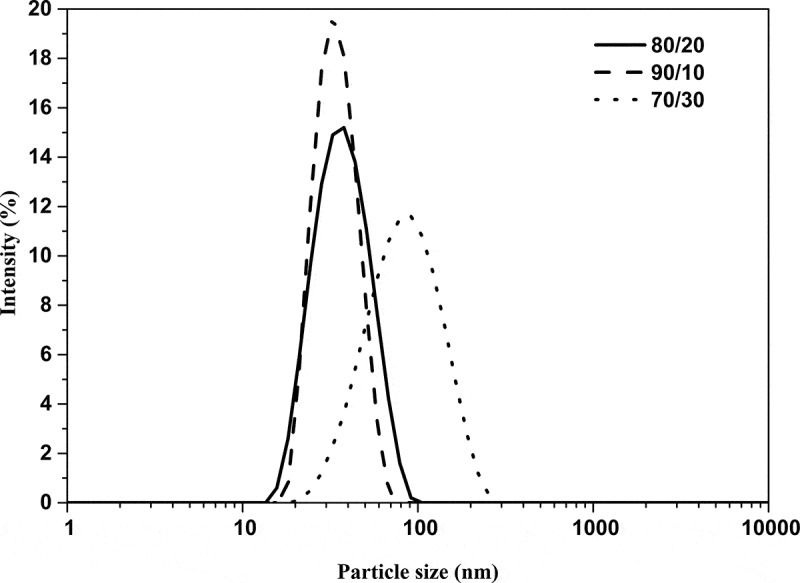
Laser light scattering graphs of latexes of samples 80/20, 90/10, and 70/30

**Figure 4.**
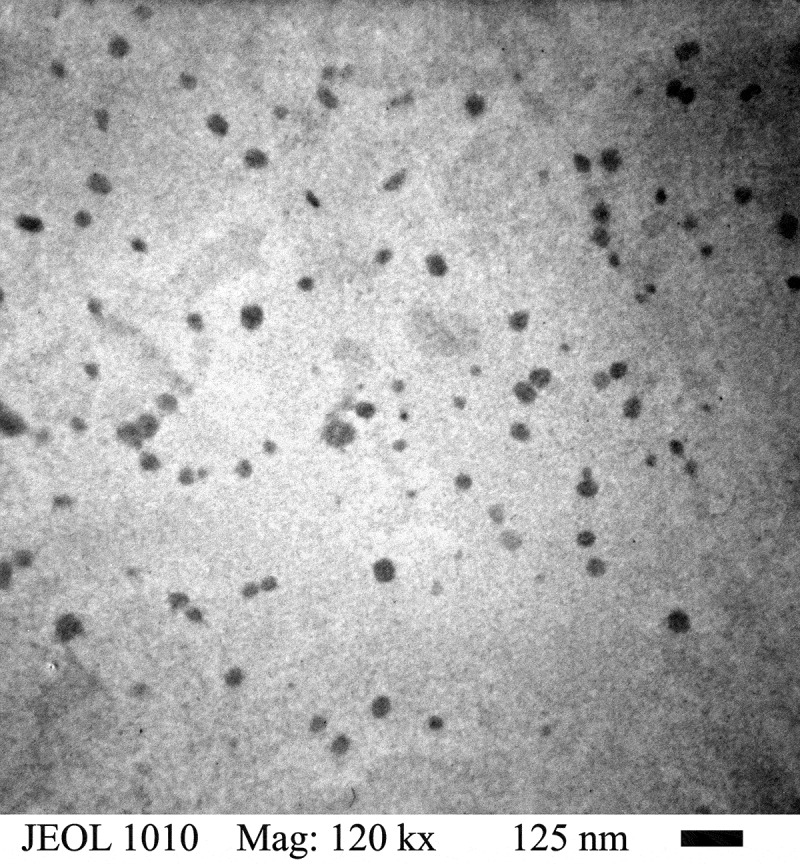
TEM of BA/PEGDMA latex particles obtained from relation 90/10% wt

**Figure 5.**
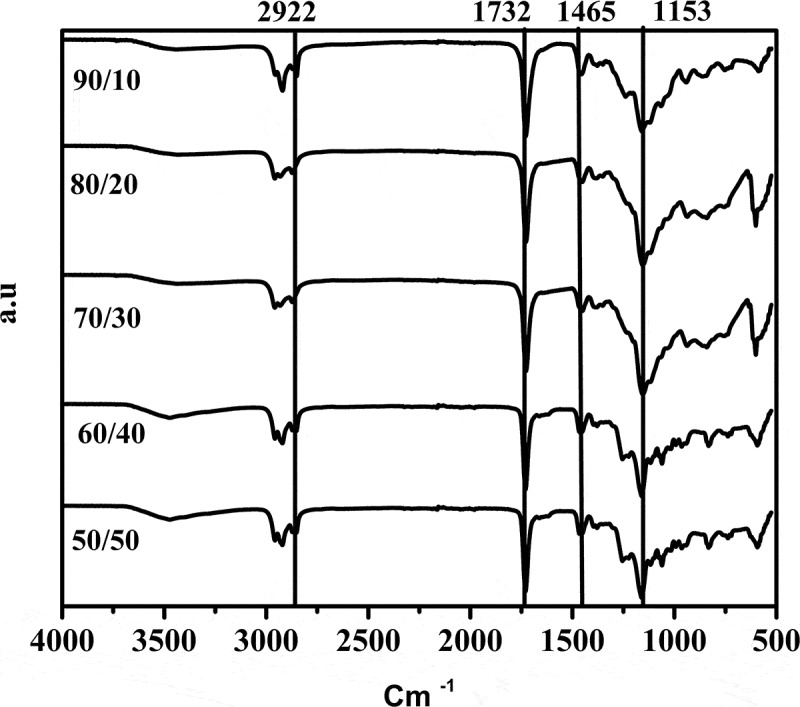
Infrared spectra of the samples

**Figure 6.**
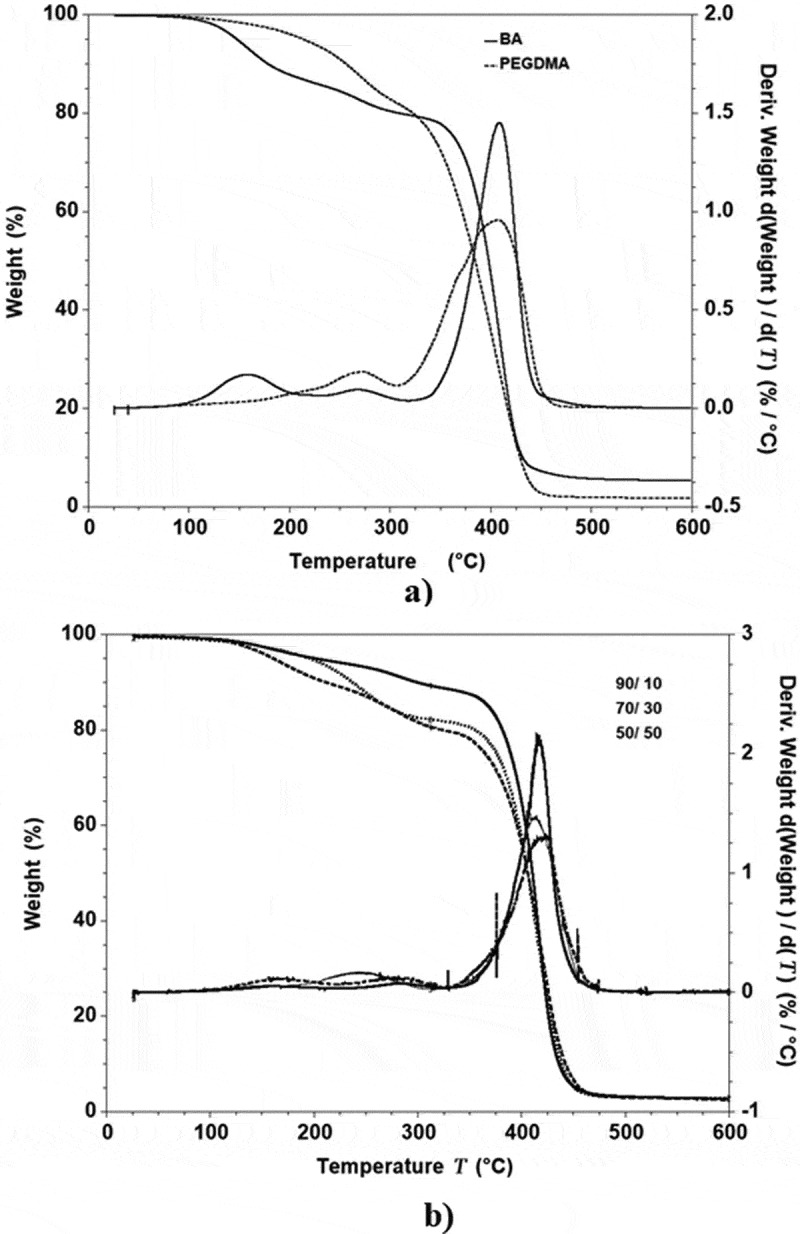
Thermal gravimetric analysis (TGA) curves of the microemulsion samples with a content of 90/10, 70/30, and 50/50% wt.BA/PEGDMA

**Figure 7.**
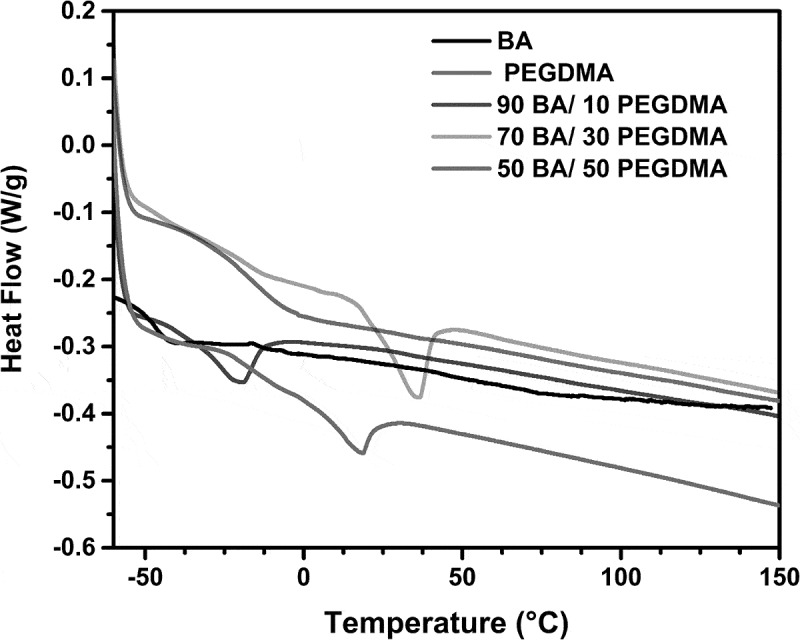
DSC scans of polymers at different concentrations of PEGDMA

Figure 2 depicts the evolution of the particle size from the latexes obtained (Dpz) as a function of conversion for all relationships. It can be seen that the ratios of 90/10, 80/20 and 70/30 produced nanometer-size polymer particles with narrow size distributions. Additionally, it can be seen that when the concentration of PEGDMA changed from 20% to 30% weight, the particle size increased in an average of 40 to 85 nm. Nonetheless, the particle size was constant throughout the reaction. This scheme suggests that particles were formed in a short period of time and that the monomer diffusion from non-reacting droplets to the reacting ones is small or negligible [23]. In regards to the distribution of particle size, it was mono-modal, as a function of conversion for relationships 90/10, PSDI = 1.3, 80/20, PSDI = 1.2 and 70/30, PSDI = 1.4. An increase in particle size from 40 to 80 nm was observed for 80/20 and 70/30, accordingly, when the BA content decreased, but the PSDI increased from 1.2 to 1,4. It is possible that the reaction fomented branched-multi-cross-linking structure of PEGDMA with BA resulting in an increase in particle size. When a concentration of PEGDMA was 40 and 50 wt.%, the particle size increased accordingly. This may be because PEGDMA shows a certain degree of water solubility and it is transferred from monomer droplets through water phase, which caused particle growth, and subsequently the instability of the system, in concordance with Zhu et al. [24]. The latexes with low PEGDMA content (≤30 wt.%) were stable and have not shown significant changes in months; the appearances of these are from bluish to semi-transparent latexes. However, for the systems with higher PEGDMA content (>30 wt.%), the latexes obtained presented gel clots, and the measurements of particle size were followed up to 8 min reaction before gel formed. The PSDI was calculated from the QLS intensity (I) distributions as follows [25]:

(1) $${\text{DP}}_{\text{N}}=\;\frac{\sum {\text{I*DP}}_{\text{Z}}}{\sum \text{I}}$$

(2) $${\text{DP}}_{\text{W}}=\frac{\;\sum \left({\text{I*Dp}}_{\text{Z}}^{3}\right)}{\sum \left({\text{I*DP}}_{\text{Z}}^{2}\right)}$$

(3) $$PSDI=\frac{DP_{W}}{Dp_{N}}$$

where I is the intensity and DP_Z_ is the average diameter of Z obtained from the equipment:

In Figure 3. The graphs exhibited a skewed distribution function, the logarithmic distribution was used for describing the particle size of the microemulsion. The results show that BA/PEGDMA microemulsions are monodisperse nanoemulsions. To confirm the particle size in Figure 4. Transmission electron microscopy (TEM) shows average diameters of copolymer particles in a range from 20 nm to approximately 40 nm, by varying the concentration of BA/PEGDMA in 90/10% wt., respectively. The spectroscopy is shown in Figure 5. FTIR spectrums obtained from the different copolymer synthesized the copolymers characteristic absorption bands are: C = O group stretching vibration appeared at 1732 cm^−1^. Wave number 1732 and 1135 cm^−1^ represent the bands at 1724 cm^−1^ and 1116 cm^−1^ [26]. These are attributed to the stretching of carbonyl in the ester and to the C-O stretching in the ether of PEGDMA macromonomer units [7,27]. The group C-H stretching vibration and the C-H in-plane bending vibration bands appeared at 2850, and 2920 cm^−1^; 1377 and 1465 cm^−1^ coincide with Wan et al. [28]. In this case, the signal for the stretching of the carbonyl group of BA is superimposed with the carbonyl signal of PEGDMA (1732 cm^−1^). But, the intensity of the bands increases with increasing PEGDMA content. Figure 6. Exhibits thermo-gravimetric (TGA), two sets of dynamic analysis have been studied. The experiments were conducted over the interval between 30°C and 600°C, until the completion of the degradation process. Figure 6(a) shows the TGA thermograms of BA and PEGDMA as homopolymers. The first stage of decomposition located between 80°C and 125°C is due to the evaporation of absorbed moisture in the copolymers. The 9.6 weight loss and 19.4% in the second stage (240°C to 300°C) resulted from the decomposition of ester group present in BA and PEGDMA [29,30]. The third stage of 300°C at 440°C shows 74.2% and 76.6% weight loss due to the decomposition of BA and PEGDMA. Figure 6(b) shows the thermogravimetric curves for the different copolymers varying their composition. In the relation of 90/10 and 70/30, it can be observed the three decomposition regions. The first stage of decomposition located between 30°C and 125°C is due to the evaporation of absorbed moisture in the film. The weight loss in stage two is interpreted as the decomposition of ester group present in the copolymers [31]. The third stage of 300°C at 500°C shows a weight loss of 87%, 78% and 80%, respectively, due to the decomposition of copolymers. These temperatures were quite close to each other, indicating that the PEGDMA did not alter the thermal stability of the copolymer. In order to investigate the thermal of BA/PEGDA copolymers, it was necessary to know the behavior of BA and PEGDMA homopolymers. The thermal analysis of homopolymers of common acrylic monomers, such as BA, has been well investigated in the literature [32]. However, the Tg behavior of PEGDMA, as a functional monomer is less frequent. Hence, a detailed analysis was conducted the DSC results on the Tg of BA/PEGDMA samples are shown in Figure 7 and Table 2. In Figure 7, in all the samples presented, shift-in-base line corresponding to Tg of BA was observed below 0°C in the temperature range from −60°C to 150°C. A homopolymer of BA has a Tg of −53°C and a PEGDMA has a Tg of 4°C (Table 2). As expected, the Tg of the copolymer increased with increasing the amount of monomer PEGDMA. In addition, the polymer is a kind of random copolymer because the polymer has only one Tg [33]. All copolymers displayed a single melting point in DSC, further supporting the formation of one copolymer [34]. In Table 2, it can be observed that the Tg value of the BA/PEGDMA copolymer increased when the content of PEGDMA increased. This can be due to the fact the PEGDMA unit increased in the copolymer and, as a consequence, the methyl group and polar group (-O-CO2-). This polar group increased the intermolecular polarity, and increased the Tg value of the copolymer. Therefore, in combination with the FTIR results (Figure 5), a possible conclusion would be that in the batch reaction, when increasing the PEGDMA unit content in the BA/PEGDMA ratio, the Tg value of the copolymer increases. According to Ding et al. [35], the addition of an acrylamide unit in the butyl methacrylate/acrylamide copolymers increased the intermolecular polarity because of the occurrence of the strong polar group (–NH2), which would also increase the Tg value of the copolymer.

**Table 2.** Glass transition temperature of copolymers

Copolymer rate of BA	Tg exp (°C)
100	−53.4
90/10	−53.1
80/20	−49.8
70/30	−45.1
60/40	−36.7
50/50	−16.1
0	4

Tg exp is glass transition temperature experimental.

## Conclusions

The microemulsion copolymerization of BA and PEGDMA was performed at various BA/PEGDMA wt.% ratios, obtaining high conversions at relatively low reaction times, with high polymerization rates. Yet, the stability of produced latexes depends on the PEGDMA contents less than 30 wt.% produce very stable latexes with low particle size, similar to those obtained by typical microemulsion polymerization; meanwhile, the PEGDMA content greater than 30 wt.% leads to unstable latexes, forming clots with high particle size. The formation of the different copolymers was confirmed by FTIR. The TGA confirmed that in the degradation process, the first stage had a loss of water at approximately 80°C and 125°C; in the second stage, it starts at around 240°C and ends at around 300°C. The weight loss during the second stage is generally interpreted as the release of the ester group. The process confirms that the ester group in the studied copolymers has been completely eliminated in the second stage of the decomposition. Finally, the third stage from 300°C to 500°C confirmed that the degradation temperatures of the copolymer were very close to each other, indicating that the PEGDMA did not alter the copolymers thermal stability. Copolymers showed single glass transition temperatures between −53.37°C and −16.58°C, depending on the composition of PEGDMA in the copolymers. Resulting in the different arrangements of units of PEGDMA along in the chain affect the thermal properties of the final copolymers.
